# Of its five acyl carrier proteins, only AcpP1 functions in *Ralstonia solanacearum* fatty acid synthesis

**DOI:** 10.3389/fmicb.2022.1014971

**Published:** 2022-09-23

**Authors:** Yu Yin, Rui Li, Wei-Ting Liang, Wen-Bin Zhang, Zhe Hu, Jin-Cheng Ma, Hai-Hong Wang

**Affiliations:** Guangdong Provincial Key Laboratory of Protein Function and Regulation in Agricultural Organisms, College of Life Sciences, South China Agricultural University, Guangzhou, Guangdong, China

**Keywords:** *Ralstonia solanacearum*, acyl carrier protein, fatty acid biosynthesis, pathogenicity, polyketide synthesis

## Abstract

The fatty acid synthesis (FAS) pathway is essential for bacterial survival. Acyl carrier proteins (ACPs), donors of acyl moieties, play a central role in FAS and are considered potential targets for the development of antibacterial agents. *Ralstonia solanacearum*, a primary phytopathogenic bacterium, causes bacterial wilt in more than 200 plant species. The genome of *R. solanacearum* contains five annotated *acp* genes, *acpP1*, *acpP2*, *acpP3*, *acpP4*, and *acpP5*. In this study, we characterized the five putative ACPs and confirmed that only AcpP1 is involved in FAS and is necessary for the growth of *R. solanacearum*. We also found that AcpP2 and AcpP4 participate in the polyketide synthesis pathway. Unexpectedly, the disruption of four *acp* genes (*acpP2*, *acpP3*, *acpP4*, and *acpP5*) allowed the mutant strain to grow as well as the wild-type strain, but attenuated the bacterium’s pathogenicity in the host plant tomato, suggesting that these four ACPs contribute to the virulence of *R. solanacearum* through mechanisms other than the FAS pathway.

## Introduction

**FIGURE 1:**
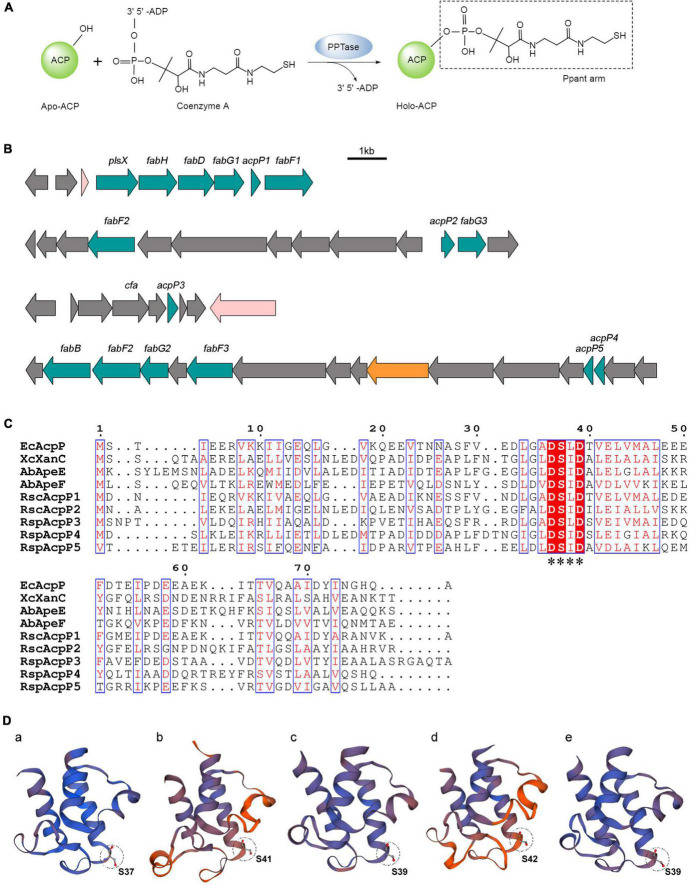
*In silico* analysis of *R*. *solanacearum* ACPs. **(A)** Posttranslational modification of acyl carrier proteins (ACPs). *Apo*-ACP was activated posttranslationally by the attachment of a Ppant arm derived from coenzyme A. The reaction was catalyzed by the 4′-phosphopantetheinyl transferase (PPTase). **(B)** Genetic organization of five *acp* genes in the *R*. *solanacearum* GMI1000 genome. **(C)** Sequence alignments of ACPs. The alignments were performed with T-Coffee (http://tcoffee.crg.cat/) and ESPript (https://espript.ibcp.fr/ESPript/ESPript/). White characters with red backgrounds are 100% identical, red characters with blue frames are similar. The conserved motif is labeled with asterisks. **(D)** Three-dimensional structure prediction of RsACPs using SWISS-MODEL (https://swissmodel.expasy.org/). The circle patterns show the conserved serine active site. **(a)** AcpP1 (template 4ihf.1.F), **(b)** AcpP2 (template 2l4b.1.A), **(c)** AcpP3 (template 3gzm.1.A), **(d)** AcpP4 (template 2 × 2b.1.A), and **(e)** AcpP5 (template 1 × 3o.1.A).

The acyl carrier proteins (ACPs) are universal, small, but flexible proteins (Prescott and Vagelos, 1972; White et al., 2005). The translation product of the *acp* gene is the inactive *apo*-form ACP. *Apo*-ACP must be converted to the active *holo*-form by 4′-phosphopantetheinyl transferase (PPTase), which transfers the 4′-phosphopantetheine (Ppant) group from coenzyme A (CoA) to a conserved serine residue in ACP with phosphoester bonding (Figure 1A; Elovson and Vagelos, 1968; Cronan and Thomas, 2009; Beld et al., 2014). Correspondingly, *holo*-ACP can be converted to *apo*-ACP by the cleavage of the Ppant arm by ACP phosphodiesterase (AcpH; Fischl and Kennedy, 1990; Thomas and Cronan, 2005). ACP belongs to a broad family of strongly related proteins with a conserved signature motif (Asp–Ser–Leu, DSL), and its structure typically consists of four α-helices (White et al., 2005). ACPs function as the carriers of acyl intermediates and are the essential cofactors of primary and secondary metabolic pathways, including those of fatty acid synthesis (FAS), polyketide synthesis (PKS), and the biosynthesis of phospholipids, glycolipids, and endotoxins (Byers and Gong, 2007). Because it plays a central role in FAS, which is essential for bacterial survival, ACP has become a target of interest for new antibacterial drugs (Sílvia et al., 2008).

*Escherichia coli* encodes only a single and essential ACP, called AcpP, which acts in the synthesis of fatty acids, phospholipids, and lipid A (Cronan and Thomas, 2009). As well as interacting with the enzymes of lipid metabolism, *E. coli* AcpP also acts as a partner of various kinds of proteins, including MdoH, IscS, SpoT, and YchM (Therisod and Kennedy, 1987; Gully et al., 2003; Battesti and Bouveret, 2006; Babu et al., 2010). It suggests that *E. coli* AcpP is also involved in membrane-derived oligosaccharide (MDO) synthesis, cysteine thiolysis, (p)ppGpp metabolism, and bicarbonate transport.

However, most bacteria have more than one ACP, and some ACPs may not affect bacterial survival. *Pseudomonas aeruginosa* has three ACPs, of which AcpP1 functions as the essential ACP in fatty acid biosynthesis and the production of N-acyl homoserine lactones (AHLs), and AcpP3 is involved in the oxidative stress response (Ma et al., 2017; Chen et al., 2018). *Enterococcus faecalis* contains two ACPs involved in fatty acid metabolism: AcpA is mainly responsible for FAS, whereas AcpB may not play a role in FAS but channels exogenous acyl groups and forms the acyl–AcpB–FabT complex, which regulates the fatty acid biosynthesis (*fab*) operon (Zhu et al., 2019). In *Xanthomonas campestris*, there is one ACP, XanC, involved in PKS and the production of the yellow pigment xanthomonadin, and the typical AcpP also plays a role in FAS (Cao et al., 2018; Chen et al., 2022). The two ACPs of *Myxococcus xanthus* and *Acinetobacter baumannii* are reportedly involved in the secondary metabolism: MxTaB and MxTaE are involved in the antibiotic TA biosynthesis, and AbApeE and AbApeF are involved in the biosynthesis of aryl polyene (APE; Paitan et al., 1999; Grammbitter et al., 2019; Lee et al., 2021). *Sinorhizobium meliloti* expresses six ACPs, of which the canonical AcpP is responsible for general FAS and three ACPs are associated with fatty acids that form lipopolysaccharides (López-Lara and Geiger, 2000). NodF is involved in the synthesis of the C16 polyunsaturated fatty acid chains in the nodulation factor mono-N-acylated chitooligosaccharide; AcpXL in the transfer of long hydroxylated fatty acids to lipid A in lipopolysaccharides; and RkpF in the synthesis of polysaccharides that are rich in 3-deoxy-D-manno-2-octulosonic acid (Demont et al., 1993; Petrovics et al., 1993; Brozek et al., 1996; Epple et al., 1998). However, the functions of the other two ACPs are still unclear (Ramos-Vega et al., 2009; Davila-Martinez et al., 2010).

*Ralstonia solanacearum* is one of the most serious soil-borne phytopathogenic bacteria (Hayward, 1991; Mansfield et al., 2012; Peeters et al., 2013). It naturally invades plants through the root tips and the cracks caused by the secondary root emergence or elongation, and subsequently reaches and fills the xylem vessels, spreading systematically and forming vascular obstructions. This clogging causes wilting symptoms in the stem and leaves, and ultimately the death of the entire plant (Vasse et al., 1995; Lowe-Power et al., 2018). During this process, a bacterial quorum-sensing (QS) signal molecule [methyl 3-hydroxypalmitate (3-OH-PAME) or methyl 3-hydroxymyristate (3-OH-MAME)] autoregulates the production of various pathogenicity related factors and secondary metabolites (Flavier et al., 1997; Kai et al., 2015).

The *R. solanacearum* GMI1000 genome encodes five putative ACPs (Figure 1B; Salanoubat et al., 2002). AcpP1 is encoded by the gene RSc1053 (now named *acpP1*), which occurs in the fatty acid biosynthesis gene (*fab*) cluster. AcpP2 is encoded by RSc0434 (now named *acpP2*), also located on the chromosome. AcpP3, AcpP4, and AcpP5 are encoded by RSp1659, RSp0370, and RSp0369, respectively (now named *acpP3*, *acpP4*, and *acpP5*, respectively), and these genes are encoded on the megaplasmid. However, the function of none of these ACPs has been reported.

In this study, we characterized these five putative ACPs and confirmed that only AcpP1 is involved in FAS and is necessary for the survival of *R. solanacearum*. We also presented evidence that AcpP2 and AcpP4 can be involved in the PKS pathway, but their specific functions in *R*. *solanacearum* require further investigation. We also found that each *acp* gene product affected the bacterial capacity for swimming motility, and the disruption of four *acp* genes (*acpP2*, *acpP3*, *acpP4*, and *acpP5*) attenuated its pathogenicity against the host plant tomato.

## Materials and methods

### Materials

Coenzyme A, fatty acids, and antibiotics were obtained from Sigma-Aldrich (St. Louis, MO, USA). Sodium [1-^14^C] acetate was provided by American Radiolabeled Chemicals, Inc. (St. Louis, MO, USA). Ni-NTA agarose was provided by Invitrogen (Shanghai, China). Takara Biotechnology (Dalian, China) provided molecular biology reagents. All other reagents were of the highest available quality. Oligonucleotide primers and sequencing were provided by Sangon Biotech Co. (Shanghai, China).

### Bacterial strains and growth conditions

The bacterial strains and plasmids used in this study are listed in Supplementary Table 1. BG medium (bacto peptone 10 g/L, glucose 5 g/L, casamino acids 1 g/L, and yeast extract 1 g/L) and NYG medium (Cao et al., 2018) were used for the growth of the *R*. *solanacearum* and *X*. *campestris* strains, respectively. Luria-Bertani (LB) broth was used for the growth of *E*. *coli*, *P*. *aeruginosa*, and *Agrobacterium tumefaciens*. RB medium (Ulrich et al., 1983) was used to test the growth of *E*. *coli acpP* mutant strain CY1877. Antibiotics were used at the following concentrations: ampicillin 100 μg/ml, kanamycin 30 μg/ml, chloramphenicol 30 μg/ml, and gentamicin 10 μg/ml for *R*. *solanacearum* or 30 μg/ml for *E*. *coli*. Isopropyl-β-D-thiogalactoside (IPTG) and L-arabinose were used at final concentrations of 1 mM, and 5-bromo-4-chloro-3-indolyl-β-D-galactoside (X-Gal) was used at a concentration of 0.1 mM.

### Recombinant DNA techniques and construction of plasmids

To clone the *R. solanacearum acp* genes, genomic DNA was extracted from *R. solanacearum* GMI1000 with the E.Z.N.A.^®^ Bacterial DNA Kit (Omega Bio-Tek, Inc., Norcross, GA, USA). The PCR products amplified from strain GMI1000 genomic DNA with *Pfu* DNA polymerase and the primers listed in Supplementary Table 2 were digested with *Nde*I and *Hin*dIII and then inserted between the corresponding sites of pET-28b, pSRK-Km, or pSRK-Gm. To generate pTac85-derived plasmids, the PCR fragments were amplified from pSRK-Gm-derived plasmids and then digested with *Nco*I and *Sal*I, the restriction sites for which had been designed into the primer sequences. After purification, the fragments were ligated into the vector pTac85. We used a similar process to construct the plasmids expressing site-directed mutant genes, except that the fragments were amplified by dividing the gene into two parts with primers carrying the mutation site. The two fragments of the gene were then purified and used for overlapping PCR. The plasmids used in this study are listed in Supplementary Table 1. The DNA fragments were confirmed with DNA sequencing by Shanghai Sangon, Inc. (Shanghai, China).

### Generation of *acp* mutant strains

To mutate the *R*. *solanacearum acp* genes, suicide plasmids were constructed with three strategies (Supplementary Figure 1). To create unmarked deletion mutants of the *acp* genes, DNA fragments (of about 600 bp) flanking the *acp* genes were amplified with the primers listed in Supplementary Table 2. The PCR products were purified and used for overlapping PCR. The resulting 1.2-kb DNA fragments were digested with *Hin*dIII and *Xba*I or *Bam*HI and inserted between the corresponding sites in pK18mobscaB. Using similar procedures, the *EcacpP* fragment and the fragments flanking *acpP1* were ligated with overlapping PCR and cloned into plasmid pK18mobsacB to construct the suicide plasmids for the *acpP1* replacement mutant. To create site-directed mutants, DNA fragments (of about 700 bp) flanking the *acpP1* mutant sites were amplified with the primers listed in Supplementary Table 2. The PCR products were purified, ligated with overlapping PCR, and cloned into pK18mobsacB.

The unmarked *acp* mutant strains were constructed with allelic exchange using suicide vector pK18mobscaB (Supplementary Figure 1) (Schäfer et al., 1994), with the procedure described by Mao et al. (2015). Briefly, all pK18mobscaB-derived plasmids were introduced into *R*. *solanacearum* by conjugal transfer from *E*. *coli* S17-1. Single-crossover integrants into the strain GMI1000 chromosome were selected based on chloramphenicol and kanamycin resistance. Cultures grown from the integrants were plated onto BG plates containing 12% sucrose to select for the loss of the vector sequences from the GMI1000 chromosome. The successful construction of the designed mutants was evaluated with PCR and the primers listed in Supplementary Table 2, and was confirmed with DNA sequencing by Shanghai Sangon, Inc. (Shanghai, China). Using similar procedures, a two gene-mutated strain was generated in the background of the single gene-mutated strain, and was then used to generate the three gene-mutated strain, which was used, in turn, to generate the four gene-mutated strain.

### Purification of *Ralstonia solanacearum* acyl carrier proteins

The pET-28b-derived plasmids were introduced into *E. coli* BL21(DE3) host cells. The ACPs with N-terminal 6 × His-tagged were expressed after the cells were induced with IPTG, and purified as described previously (Chen et al., 2022). To obtain *apo*-form ACPs, *E. coli* strain YY121 expressing *E. coli* AcpH was also cultured and the protein expressed in LB broth. The cells expressing ACPs and AcpH were harvested and lysed by sonication in AcpH reaction buffer [50 mM Tris–HCl (pH 8.8), 25 mM MgCl_2_, 1 mM dithiothreitol (DTT), 0.2 mM MnCl_2_; Cronan and Thomas, 2009]. Samples of *holo*-ACPs were converted to *apo*-ACPs with AcpH supernatant (the volumetric ratio of ACP solution to AcpH solution was 2:1). After incubation at 37°C for 4 h, the mixture was applied to Ni NTA Beads 6FF (Smart-Lifesciences Inc., China), according to the general protocol of the manufacturer. The eluted ACP was precipitated by the addition of trichloroacetic acid at a final concentration of 8%, and was then redissolved and dialyzed twice in AcpS reaction buffer [50 mM Tris-HCl (pH 8.8), 10 mM MgCl_2_, 5 mM DTT; Cronan and Thomas, 2009].

The plasmids pET-acpS, pET-Sfp, and pYFJ84 were introduced into *E. coli* BL21(DE3) host cells for the expression of *E. coli* AcpS, *Bacillus subtilis* PPTase Sfp, and *Vibrio harveyi* acyl-ACP synthetase (AasS) proteins, respectively. These proteins were also purified as described previously (Jiang et al., 2006; Chen et al., 2022).

### Phosphopantetheinylatation and acylation of acyl carrier proteins *in vitro*

The phosphopantetheinylatation and acylation assays were adapted from those of Ma et al. (2017). Briefly, the reaction mixture for phosphopantetheinylatation contained 50 mM Tris–HCl (pH 8.8), 10 mM MgCl_2_, 1 mM DTT, 1 mM CoA, 50 μM *apo*-ACP, and 1 μM AcpS or Sfp, and was incubated at 37°C for 1 h. The reaction products were resolved with conformation-sensitive gel electrophoresis on 17.5% polyacrylamide gels containing 2.5 M urea, optimized for separation.

*Vibrio harveyi* AasS was used to acylate *holo*-ACP (Jiang et al., 2006). The reaction mixtures for acylation contained 100 mM Tris–HCl (pH 8.0), 10 mM MgCl_2_, 10 mM ATP, 5 mM DTT, 0.5 mM hexanoic acid or dodecanoic acid, 25 μM *holo*-ACP (*apo*-ACP was incubated with PPTase), and 2 μM AasS, and were incubated at 37°C for 1 h. The reaction products were resolved on 17.5% polyacrylamide gels containing 4 M urea.

### Analysis of phospholipid fatty acids

The *R*. *solanacearum* strains were cultured at 30°C in BG medium overnight, transferred to 5 ml of BG medium, and grown from an optical density at a wavelength of 600 nm (OD_600_) of 0.2 to an OD_600_ of 0.7–1.0 with or without additional sodium [1-^14^C] acetate (final concentration, 1 μCi/ml). To assay *de novo* FAS, the sodium [1-^14^C] acetate-labeled cells were pelleted and lysed with methanol-chloroform (2:1) solution, and the fatty acid methyl esters were extracted as previously described (Zhu et al., 2019). The labeled fatty acids were analyzed with thin-layer chromatography (TLC) and quantified with phosphorimaging. To assay the phospholipid compositions, fatty acid methyl esters were synthesized with sodium methoxide and extracted with petroleum ether, as described previously (Zhu et al., 2010), and were analyzed with GC–MS.

### Assay of quorum-sensing signal molecule

An assay of the QS signal molecule was performed as described previously (Guo et al., 2018). *P. aeruginosa* strains were grown to an OD_600_ of 1.0, and the cells were removed by centrifugation at 4°C. The QS signal molecule HSL was twice extracted from 10 ml of supernatant from each sample with an equal volume of ethyl acetate. The organic extract was concentrated to dryness with a nitrogen bubbler, and the residue was resuspended in 100 μL of ethyl acetate. The biosensor *A*. *tumefaciens* NT1 (*traR*, *tra*::*lacZ749*) was grown in LB broth to an OD_600_ of 1.0 and then diluted to an OD_600_ of 0.1 to cover the ME medium plate (0.2 g/L MgSO_4_⋅7H_2_O, 10 g/L K_2_HPO_4_, 2 g/L C_6_H_8_O_7_⋅H_2_O, 3.5 g/L NaNH_4_HPO_4_⋅4H_2_O, pH 7.0) supplemented with X-Gal and IPTG. The extracts of the *P*. *aeruginosa* strains were dotted onto the ME plate described above. After overnight incubation at 30°C, a blue halo was observed when N-(3-oxo-dodecanoyl)-L-homoserine lactone (3-oxo-C12-HSL) was produced (Piper et al., 1993).

### Extraction and quantification of xanthomonadins

The pigments were extracted from *X*. *campestris* with a previously described procedure (Cao et al., 2018). Briefly, *X*. *campestris* was grown to stationary phase (OD_600_ = 1.0) in NYG medium at 30°C. The cells from 10 ml of each culture were collected by centrifugation. The pigments were extracted by shaking with 1 ml of methanol for 5 min. The amounts of xanthomonadin pigment produced were expressed as the absorbance (OD_445_) of the crude pigment extracts.

### RNA extraction, reverse transcription PCR, and quantitative real-time PCR

*Ralstonia solanacearum* strain GMI1000 was grown in BG medium at 30°C. The total RNA was extracted from the cells in 0.5 ml of culture from each growth phase with a E.Z.N.A.^®^ Bacterial RNA Kit (Omega Bio-Tek, Inc.). Once extracted, 1 μg of total RNA was reverse transcribed with the PrimeScript™ RT reagent Kit with gDNA Eraser (Takara Bio Inc.). Quantitative real-time PCR (qPCR) was performed with the 2 × RealStar Green Fast Mixture (GenStar). Relative quantitation was done by the comparative cycle threshold (ΔΔCT) method using the endogenous internal control 16S rDNA and *gryB* (DNA gyrase subunit B; Castillo and Greenberg, 2007) for sample normalization. The primers used in reverse transcription PCR (RT-PCR) and qPCR are listed in Supplementary Table 2.

### Swimming motility assay

The motility of *R*. *solanacearum* was assayed on semisolid motility medium containing 1% (wt/vol) bacterial peptone and 0.3% (wt/vol) agar (BD). A 2 μL aliquot of cell suspension containing 1.0 × 10^8^ CFU/ml was added to the center of the plate and cultured at 30°C. The diameters of migration were measured at 72 h postinoculation.

### Pathogenicity tests

The bacterial virulence experiment using drenching infection assays was adapted from Morel et al. (2018). The roots of each 4-week-old tomato plant (*Lycopersicon esculentum* cv. Moneymaker) were partly cut, and were inoculated by pouring 20 ml of fresh bacterial suspension containing 5 × 10^9^CFU of *R*. *solanacearum* onto the soil surrounding the plants. Once infected, the plants were incubated in a growth chamber (28°C day/27°C night, 85% relative humidity, 12 h light), and wilting symptoms were recorded daily on a severity rating visual scale, from 0 (no wilt) to 4 (death). Each strain tested was assessed for wilting in three independent experiments (15 plants each). The severity of the infection was expressed with the disease index (DI), which was calculated with the formula: DI = ∑(severity rating × number of plants in that rating)/(total number of plants × 4).

### Data confirmation and statistical analysis

Experiments were repeated three times to confirm their reproducibility. All results were analyzed with GraphPad Prism 8 and are presented as means ± standard deviations (SD). The statistical significance of the difference between two measurements was determined with Student’s *t-*test. Results were considered statistically significant at *P* < 0.05.

## Results

### *In silico* analysis of *Ralstonia solanacearum* acyl carrier proteins

The multiple alignments of ACP amino acid sequences were performed with T-Coffee and ESPript (Figure 1C). AcpP1, encoded within a *fab* gene cluster, showed high identity with *E. coli* AcpP (68.35%), and AcpP3 and AcpP5 are also similar to *E. coli* AcpP (35.63% and 29.63% identity, respectively; Supplementary Table 3). The *acpP2* gene is located in a cluster similar to the *pig* cluster, which is responsible for xanthomonadin biosynthesis in *X. campestris* (Cheng et al., 2012; Cao et al., 2018), and AcpP2 is 48.31% identical to XcXanC encoded by *acpC* in the *pig* cluster. The megaplasmid genes *acpP4* and *acpP5* are located in a cluster syntenic to the *ape* gene cluster, which is responsible for the biosynthesis of APE pigments in *A. baumannii* (Grammbitter et al., 2019; Lee et al., 2021). Two genes are located in the *ape* cluster, encoding AbApeE and AbApeF, and AcpP4 is 44.09% identical to AbApeE and AcpP5 is 41.46% identical to AbApeF. The alignment also showed that all ACPs contain the conserved serine catalytic triad Asp–Ser–Leu (DSL).

The prediction of the three-dimensional structures of the *R*. *solanacearum* ACPs with the SWISS-MODEL server showed that the topologies of the five ACPs are similar to that of *E. coli* AcpP (Figure 1D; White et al., 2005). They all have four helices (except AcpP2, which has no α3 helix) and the DSL motif at the end of helix α2 (Figure 1D and Supplementary Figure 2; Rock and Cronan, 1979; Roujeinikova et al., 2002; White et al., 2005). The results of a bioinformatic analysis imply that these five putative proteins in *R. solanacearum* are ACPs, among which AcpP1 and AcpP3 probably function in FAS and AcpP2, AcpP4, and AcpP5 in PKS.

### *Ralstonia solanacearum* acyl carrier proteins are phosphopantetheinylated *in vitro*

In general, *apo*-ACP requires posttranslational modification for its activation (Figure 1A). To determine whether the five ACPs of *R. solanacearum* are functional, the *apo*-forms of AcpP1, AcpP2, AcpP3, and AcpP5 were isolated to examine their phosphopantetheinylatation. These *apo*-ACPs were purified as described in section “Materials and methods.” Curiously, attempts to isolate AcpP4 were unsuccessful, despite the optimization of its codons, varying the expression time (4–12 h) and temperature (18–37°C), its co-expression with the different protein partners (TrxA, MBP, and GST), and its fusion expression with different protein tags (MBP, GB1, Fh8, SUMO, and TrxA).

**FIGURE 2:**
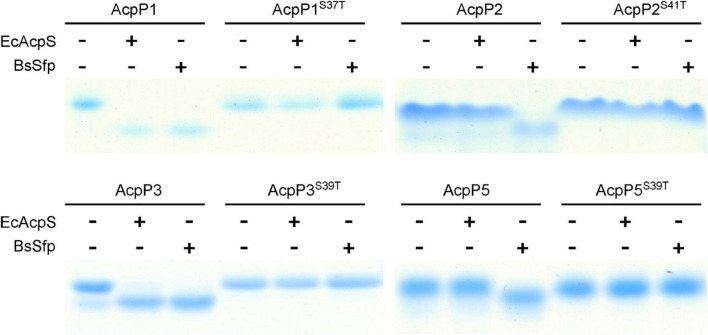
Four *R*. *solanacearum* ACPs were phosphopantetheinylated *in vitro*. The reaction mixture contained ACP, CoA, and *E*. *coli* AcpS or *B*. *subtilis* Sfp in AcpS reaction buffer. The *apo*-ACPs tested as the reaction substrate are given above each lane.

AcpS-type PPTases primarily act on the ACPs of the FAS pathway, whereas Sfp-type PPTases have a broader substrate range, and can modify the ACPs involved in the secondary or primary metabolism (Quadri et al., 1998; Flugel et al., 2000; Mootz et al., 2001; Beld et al., 2014). Therefore, *E. coli* AcpS and *B. subtilis* Sfp were used to examine the modification of the *R. solanacearum* ACPs. After incubation with AcpS or Sfp and CoA, the migration of AcpP1 and AcpP3 changed significantly, indicating that AcpP1 and AcpP3 are activated from *apo*-ACP to *holo*-ACP by these two types of PPTase (Figure 2). The migration of AcpP2 and AcpP5 changed in the presence of Sfp, but not in the presence of AcpS, indicating AcpP2 and AcpP5 are only activated by Sfp-type PPTases. These data show that these four *R. solanacearum* ACPs can be phosphopantetheinylated, and that AcpP1 and AcpP3 may be involved in FAS, whereas AcpP2 and AcpP5 may be involved in the secondary metabolism.

According to previous research, ACPs contain the conserved DSL motif, in which the serine is the active site through which the Ppant group is linked, and mutation at that residue causes protein loss of function (White et al., 2005; De Lay and Cronan, 2007). Therefore, the *apo*-forms of site-directed mutant proteins in which the Ser in DSL was replaced with Thr were also tested. After incubation with CoA, the migration of the four mutant proteins did not shift in the presence of either AcpS or Sfp, indicating that the mutant ACPs were not activated by these two types of PPTase (Figure 2), confirming that the Ser in the DSL motif is the key active site of the ACPs, as previously reported.

### Only the *acpP1* gene is necessary for *Ralstonia solanacearum* survival

**FIGURE 3:**
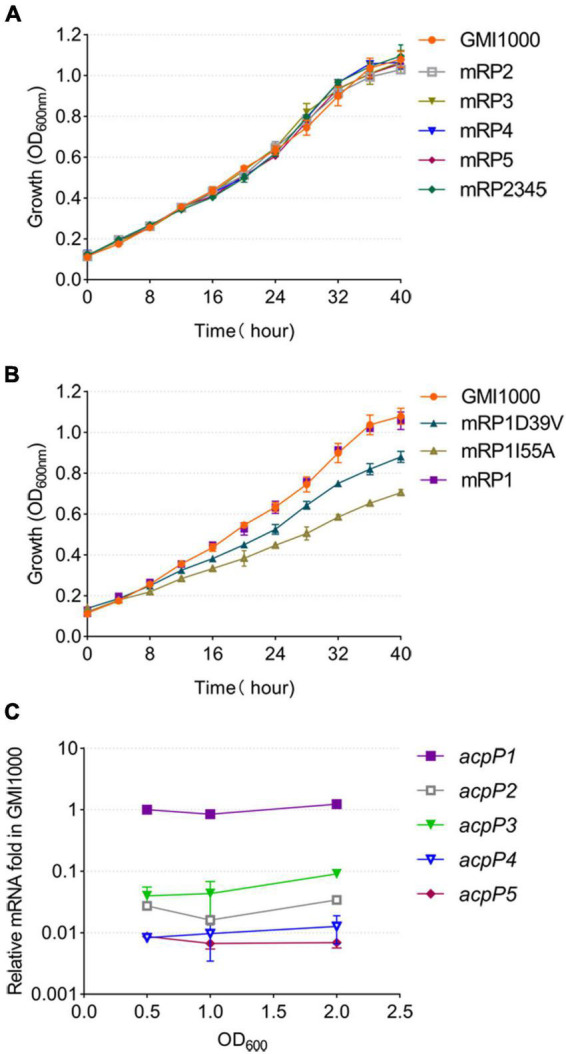
Only the AcpP1 affects the growth of *R*. *solanacearum*. **(A,B)** Growth of the strains in BG medium at 30°C. GMI1000, wild-type strain; mRP1D39V, mutant in which Asp39 of AcpP1 is altered to Val; mRP1I55A, mutant in which Ile55 of AcpP1 is altered to Ala; mRP1, mutant in which the *acpP1* gene is replaced with *E. coli acpP*; mRP2, mutant lacking *acpP2* gene; mRP3, mutant lacking *acpP3* gene; mRP4, mutant lacking *acpP4* gene; mRP5, mutant lacking *acpP5* gene; mRP2345, mutant lacking *acpP2*, *acpP3*, *acpP4*, and *acpP5* genes. **(C)** Expression of the five *acp* genes in *R*. *solanacearum* wild-type strain GMI1000. Expression level of the *acpP1* gene in log phase (OD_600_ = 0.5) was set to 1.

To determine the function of each ACP, the *acpP2*, *acpP3*, *acpP4*, or *acpP5* gene was deleted individually to generate mutant strain mRP2, mRP3, mRP4, or mRP5, respectively. A mutant lacking all four *acp* genes (mRP2345) was also generated. The growth of these mutants in the rich BG medium (Figure 3A) or minimal M63 medium (data not shown) was no different from that of the wild-type strain. In contrast, the *acpP1*-deletion mutant could not be isolated, suggesting that *acpP1* is an important housekeeping gene in *R. solanacearum*.

Based on the importance of *acpP1* and the similarity of the protein sequences encoded by *acpP1* and *E. coli acpP*, the *E. coli acpP* gene was used to replace the *acpP1* gene in *R. solanacearum*. The mutant strain in which the *acpP1* gene was replaced with *E. coli acpP* (designated mRP1) was generated with this strategy. Previous research has shown that some single-site mutations of *E. coli* AcpP impair, but do not entirely destroy, the activity of the protein (De Lay and Cronan, 2007). Therefore, we attempted to site-specifically mutate the *acpP1* gene with allelic replacement using the plasmid pK18mobscaB (Supplementary Figure 1). Two site-directed mutants were obtained: mRP1D39V (Asp39 mutated to Val) and mRP1I55A (Ile55 mutated to Ala). Although the replacement of the *acpP1* gene with *E. coli acpP* did not affect the growth of the strain, the site-specific mutation of AcpP1 at Asp39 or Ile55 significantly impaired the growth of the strain (Figure 3B). All these data confirm that *acpP1* is essential for *R*. *solanacearum* viability.

Using qPCR to quantify the transcription levels of all the *acp* genes in the *R. solanacearum* wild-type strain, we found that *acpP1* is highly and stably expressed throughout its growth stages, whereas the expression of the other four *acp* genes was more than 10-fold lower than that of *acpP1* (Figure 3C). These results indicate that *acpP1* is highly expressed and required for the growth of *R. solanacearum*, and that mutations at certain sites result in significant growth retardation.

### Only AcpP1 functions in fatty acid biosynthesis

**FIGURE 4:**
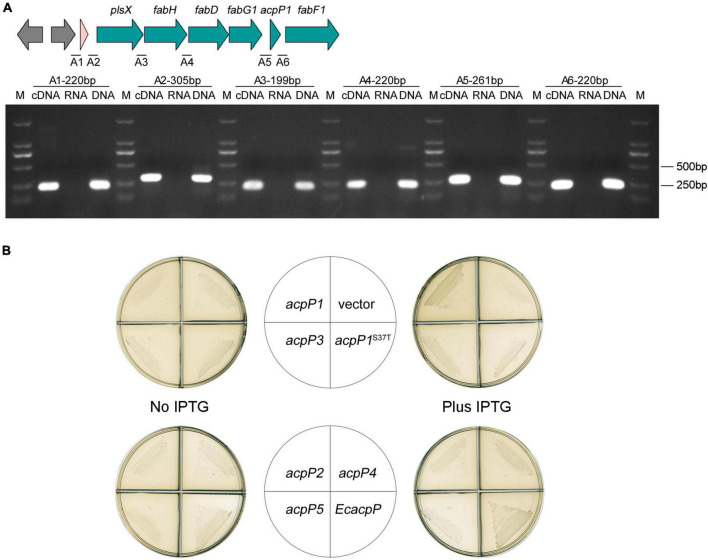
Only AcpP1 partially restored the growth of *E*. *coli acpP-*mutant strain. **(A)** RT-PCR of the intergenic regions. cDNA, RNA, or DNA from the GMI1000 strain was used as the template for PCR. The intergenic regions and lengths are marked above each lane. M: DNA marker DL2000. **(B)** Complementation of *E*. *coli acpP* mutant strain CY1877 with *R*. *solanacearum acp* genes. Strains CY1877 carrying IPTG-inducible expression vector pTac85 expressing an *acp* gene (as shown in the middle panels) were cultured on RB plates for 24 h. Left panels showed RB medium without the supplement of IPTG; right panels showed RB medium supplemented with IPTG; vector, pTac85.

In the genome of *R. solanacearum* GMI000, *acpP1* is located in the canonical *fab* gene cluster, which includes *plsX*, *fabH*, *fabD*, *fabG1*, and *fabF1* (Cheng et al., 2012; Feng et al., 2015; Mao et al., 2015). With a RT-PCR analysis of the intergenic regions, we showed that the *acpP1* gene is cotranscribed with upstream and downstream genes and is located within a *fab* gene cluster (Figure 4A). This confirms that the expression of AcpP1 is closely related to the expression of FASII enzymes.

Because *E. coli* AcpP acts in FASII, the *E. coli acpP* mutant strain CY1877, which requires arabinose to induce *E. coli acpP* expression for normal growth, was used to test the functions of the *R. solanacearum acp*-encoded proteins (Ma et al., 2017). The results showed that the overexpression of *acpP1*, induced with IPTG, partially restored CY1877 growth (Figure 4B), whereas the overexpression of the other *acp* genes failed to support the growth of the *E. coli* mutant, indicating that only *R. solanacearum* AcpP1 can functionally replace *E. coli* ACP.

**FIGURE 5:**
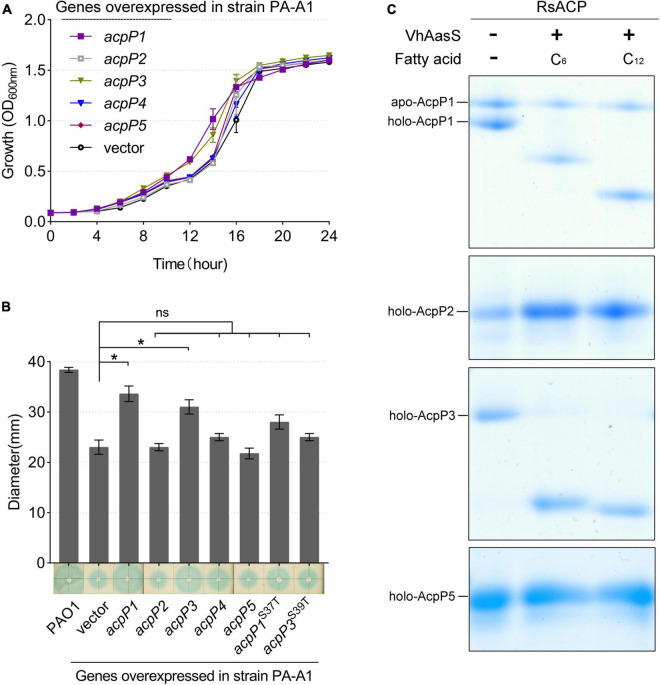
Identification of *R*. *solanacearum* AcpPs *in vivo* and *in vitro*. **(A)** Growth of *P. aeruginosa acpP1*-mutant strain PA-A1 harboring the *R. solanacearum acp* genes. Overexpression of *R*. *solanacearum acp* genes did not affect strain growth. **(B)** Detection of 3-oxo-C_12_-HSL signals produced by the complementation of strain PA-A1. The QS signal molecules were extracted as described previously and were dotted onto a ME medium plate that was supplemented with X-Gal and IPTG and covered with fresh *A*. *tumefaciens* NT1 (*traR*, *tra*::*lacZ749*; Guo et al., 2018). Student’s *t*-test was used to analyze the difference between PA-A1 carrying the empty vector and PA-A1 overexpressing the *acp* genes. *Indicates significant difference (*P* < 0.05). *^ns^*Indicates no significant difference (*P* > 0.05). **(C)** The *R*. *solanacearum holo*-AcpPs were acylated by VhAasS *in vitro*. The reaction mixtures contained *holo*-ACP, ATP, hexanoic acid or dodecanoic acid, and *V. harveyi* AasS.

Next, the *R. solanacearum acp* genes were overexpressed in *P. aeruginosa* mutant strain PA-A1, in which the chromosomal *acpP1* gene was replaced with *E. coli acpP*, impairing QS signal production (Ma et al., 2017). The fatty-acid-derivative QS signal molecule, 3-oxo-C12-HSL, produced by *P. aeruginosa* PA-A1 was tested with the biosensor *A*. *tumefaciens* NT1 (*traR*, *tra*::*lacZ*749), which produces a blue halo in response to acyl homoserine lactones (acyl-HSLs; Piper et al., 1993). The growth of *P. aeruginosa* PA-A1 derivatives carrying the plasmid pSRK-Gm encoding the *R. solanacearum acp* genes did not differ from that of PA-A1, but the blue halo around PA-A1 carrying *acpP1-* or *acpP3*-encoded plasmids was larger than that around the PA-A1 strain carrying the empty vector (Figures 5A,B). These results suggest that AcpP1 functions in FAS, whereas AcpP3 seemed to be acylated. To confirm these findings, we tested the acylation of *R. solanacearum* ACPs using *V. harveyi* acyl-ACP synthetase (AasS) *in vitro* (Byers, 1989). After incubation of the *R. solanacearum holo*-ACPs with hexanoic acid or dodecanoic acid, ATP, and VhAasS, the migrations of *holo*-AcpP1 and *holo*-AcpP3 were altered, showing that these two *holo*-ACPs were acylated by VhAasS to carry a fatty acid chain (Figure 5C). In contrast, there was no change in the migrations of *holo*-AcpP2 and *hol*o-AcpP5.

**FIGURE 6:**
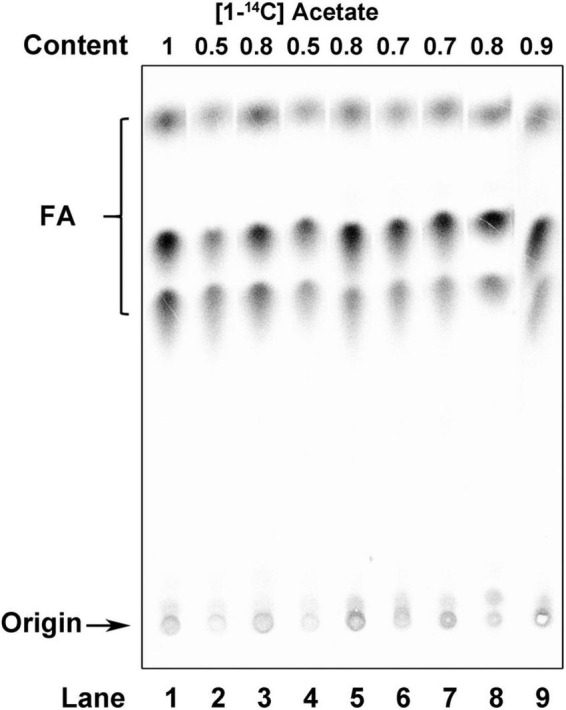
Analysis of *de novo* fatty acid synthesis in *R*. *solanacearum* strains. Argentation thin-layer chromatographic analysis of sodium [1-^14^C] acetate-labeled *R*. *solanacearum* strains, as described in section “Materials and methods.” Lane 1, wild-type strain; lane 2, mutant in which Asp39 of AcpP1 was mutated to Val; lane 3, mutant in which the *acpP1* gene was replaced with *E. coli acpP*; lane 4, mutant in which Ile55 of AcpP1 was mutated to Ala; lane 5, mutant lacking genes *acpP2*, *acpP3*, *acpP4*, and *acpP5*; lane 6, mutant lacking gene *acpP2*; lane 7, mutant lacking gene *acpP3*; lane 8, mutant lacking gene *acpP4*; lane 9, mutant lacking gene *acpP5*. The numbers above the lanes give the relative incorporation value for each lane.

To determine whether ACPs function in the FAS of *R. solanacearum*, the capacity of the *acp* mutants for FAS was determined by measuring their *de novo* FAS as the incorporation of [1-^14^C] acetate into their membrane phospholipids. The incorporation of [1-^14^C] acetate into the membrane phospholipids of the two *acpP1* mutants, mRP1D39V and mRP1I55A, was twofold lower than that in the wild-type strain (Figure 6, lanes 2 and 4). However, neither single gene deletion mutants nor the quadruple gene deletion mutant of the other four *acp* genes affected the fatty acid biosynthesis of *R. solanacearum* (Figure 6, lanes 5–9). We also analyzed the fatty acid composition of the *R. solanacearum* strains with gas chromatography-mass spectrometry (GC–MS). All *R. solanacearum* strains had similar fatty acid profiles (Supplementary Table 4). These results confirm that only AcpP1 is involved in fatty acid biosynthesis.

### AcpP2 and AcpP4 participate in polyketide synthesis

**FIGURE 7:**
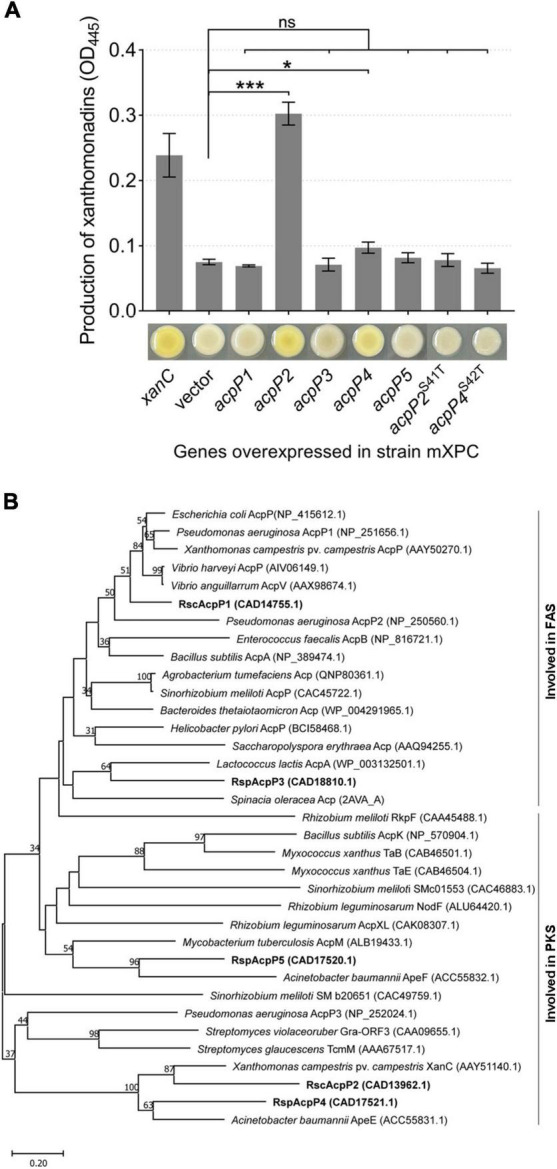
AcpP2 and AcpP4 participate in polyketide synthesis. **(A)** AcpP2 and AcpP4 restored the production of xanthomonadin in the *X*. *campestris xanC* mutant mXPC. Pigments were extracted with methanol from *X*. *campestris* using previously described procedures (Cao et al., 2018). Student’s *t*-test was used to compare mXPC carrying the empty vector and mXPC overexpressing the *acp* genes. Significant differences are indicated as ***P < 0.001 and *P < 0.05. **(B)** Neighbor-joining phylogenetic analysis of ACPs. The neighbor-joining tree was created with MEGA 11, with 1,000 bootstrap replicates. Proteins are shown with their NCBI (https://www.ncbi.nlm.nih.gov/) accession numbers in parenthesis. The RsACPs clustered into two regions, reflecting their involvement in fatty acid synthesis (FAS) or polyketide synthesis (PKS).

According to the results described above, AcpP2, AcpP4, and AcpP5 are not involved in FAS, and genes *acpP2*, *acpP4*, and *acpP5* are located in gene clusters related to the synthesis of APEs (Cao et al., 2018; Grammbitter et al., 2019). Therefore, we inferred that AcpP2, AcpP4, and AcpP5 are involved in PKS. In *X. campestris*, *xanC* encodes the ACP XanC, which is essential for the biosynthesis of xanthomonadin, a yellow APE pigment (Cao et al., 2018). Therefore, the *X. campestris xanC*-deletion mutant mXPC, which had lost the ability to synthesize yellow pigment, was constructed and used to examine whether *R. solanacearum* ACPs are involved in the PKS pathway. The overexpression of *acpP2* or *acpP4* from a plasmid pSRK-Km in the mutant mXPC restored xanthomonadin production, but the strain failed to produce xanthomonadin when the genes *acpP1*, *acpP3*, and *acpP5* were overexpressed in the mXPC mutant (Figure 7A). These results show that AcpP2 and AcpP4 can act as ACPs in PKS and indicate that AcpP4 can be phosphopantetheinylated *in vivo*. Moreover, the strains expressing the site-specifically mutated genes *acpP2*^S41T^ or *acpP4*^S42T^ also did not produce yellow pigment, indicating that AcpP4, like AcpP2, has the typical active-site characteristics (Figure 7A).

A phylogenetic analysis of the *R. solanacearum* ACPs and ACPs from different species, using the neighbor-joining algorithm in the molecular evolutionary genetics analysis software MEGA 11 (Tamura et al., 2021), was performed. On a phylogenetic tree constructed from the amino acid sequence alignment, these ACPs clustered in two major groups, involved in either FAS or PKS (Figure 7B). AcpP1 and AcpP3 clustered in the group involved in FAS, whereas AcpP2, AcpP4, and AcpP5 clustered with the other group. These data further support the inference that AcpP2 and AcpP4 are involved in the synthesis of an unknown secondary metabolite in *R. solanacearum*.

### *Ralstonia solanacearum* mutant mRP2345 showed impaired virulence in tomato

**FIGURE 8:**
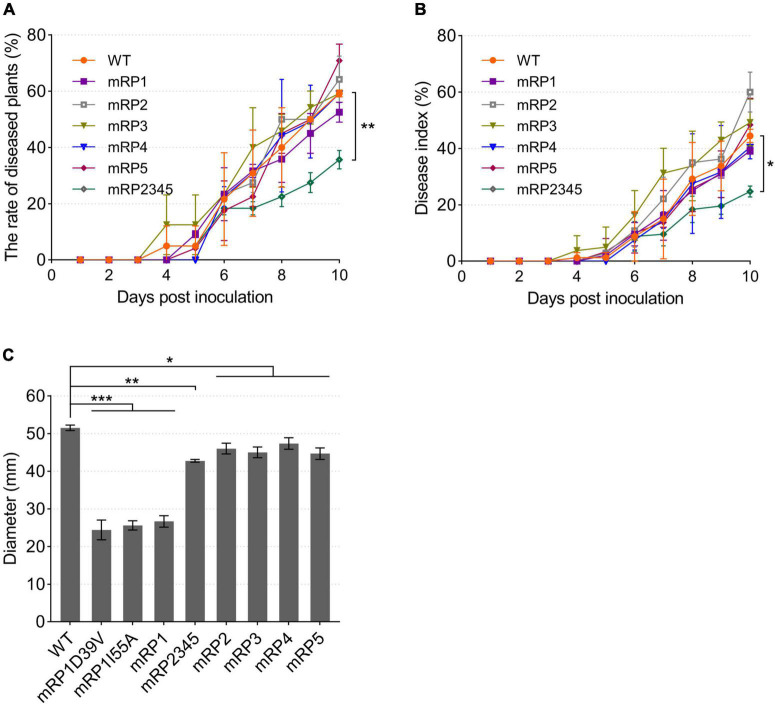
Pathogenicity and swimming motility of *R*. *solanacearum* strains. **(A,B)** Mutant mRP2345 showed significantly reduced virulence in tomato plants. Fresh cultures of the strains were used to infect tomato plants. Once infected, the numbers of diseased plants (left panel) and the disease severity rating for each plant (right panel) were recorded daily. The rate of diseased plants is the ratio of the number of diseased plants to the total number of plants. The disease index (DI), DI = ∑(severity rating × number of plants in that rating)/(total number of plants × 4). **(C)** The swimming motility patterns of the *R*. *solanacearum* strains were assessed on semisolid motility medium. WT, wild-type strain GMI1000; mRP1, mutant in which the *acpP1* gene is replaced with *E. coli acpP*; mRP2, mutant lacking gene *acpP2*; mRP3, mutant lacking gene *acpP3*; mRP4, mutant lacking gene *acpP4*; mRP5, mutant lacking gene *acpP5*; mRP2345, mutant lacking genes *acpP2*, *acpP3*, *acpP4*, and *acpP5*; mRP1D39V, mutant in which Asp39 of AcpP1 is mutated to Val; mRP1I55A, mutant in which Ile55 of AcpP1 is mutated to Ala. Significant differences are indicated as ***P < 0.001, **P < 0.01, and *P < 0.05.

To investigate the effects of these ACPs on the pathogenicity of *R. solanacearum*, tomato plants were infected by drenching the soil with the *acp* gene mutant strains. The strains caused plant wilting symptoms on days 4–6 after inoculation. By day 10, all strains caused wilting in more than 50% of the plants, except the four-gene deletion mutant mRP2345, which caused only 35.56% of the plants to develop the disease (Figure 8A). The disease severity index of mRP2345 (24.44%) was also significantly lower than that of the wild-type strain (44.44%; Figure 8B), indicating that the deletion of the four *acp* genes significantly reduced the virulence of *R. solanacearum*.

The pathogenicity related factors of the *R. solanacearum acp* mutant strains were also evaluated. First, semisolid plates were used to test the swimming motility of *R. solanacearum*. Compared with wild-type strain GMI1000, all the mutants formed smaller motility patterns, and the colonial patterns formed by the three *acpP1* mutants were only half the size of the wild-type strain pattern (Figure 8C). The biofilms, extracellular cellulases, and extracellular polysaccharides produced by the *R. solanacearum* mutants were also tested (Supplementary Figure 3). However, these phenotypes were not significantly impaired in mutant mRP2345. Therefore, the mechanisms of action of AcpP2, AcpP3, AcpP4, and AcpP5 in the virulence of *R. solanacearum* remain to be clarified.

## Discussion

The *R. solanacearum* GMI1000 genome contains five open reading frames, RSc1053, RSc0434, RSp1659, RSp0370, and RSp0369, that encode putative ACPs, AcpP1, AcpP2, AcpP3, AcpP4, and AcpP5, respectively. We confirmed that these ACPs are successfully phosphopantetheinylated and identified the active site at which the Ppant group is linked in these ACPs. Among the five *acp* genes, *acpP1* is most strongly transcribed, suggesting that *acpP1* is a housekeeping *acp* gene. *acpP1* is also cotranscribed with the *fab* cluster and could not be deleted, demonstrated that AcpP1 is necessary for the survival of *R. solanacearum*. Therefore, like *Burkholderia cenocepacia* J2315 ACP, the mutation of which reduces its ability to colonize and kill nematodes, *R. solanacearum acpP1* can also be considered a potential target for anti-infection drugs (Sílvia et al., 2008). As expectedly, two site-specific mutations in AcpP1 (D39V and I55A) impaired the growth and FAS of *R. solanacearum*. Asp39 is one of the conserved residues where AcpS binds, and previous research has shown that *E*. *coli* became temperature-sensitive when Asp39 of AcpP mutated to Val (De Lay and Cronan, 2007). Ile55 is also a key residue that interacts with the residues from the helices to provide space for the acyl chain and also interacts with FAS proteins (Roujeinikova et al., 2002, 2007). A previous study has shown that *E*. *coli* AcpP^I54A^ can be phosphopantetheinylated efficiently (De Lay and Cronan, 2007). Therefore, we infer that the mutant mRP1I55A grew slowly because AcpP1^I55*A*^ affected the space for the accepting acyl chain or impaired the interaction with *R*. *solanacearum* FAS proteins or acyl chains.

The expression of *R. solanacearum acpP3* failed to restore the growth of the *E. coli acpP*-mutant strain CY1877, and the mutation of *acpP3* did not affect fatty acid biosynthesis in *R. solanacearum.* Moreover, a mutant in which *acpP3* functionally replaced *acpP1* could not be generated. All these data indicate that AcpP3 is not involved in the FAS of *R. solanacearum.* Furthermore, AcpP3 does not affect the incorporation of exogenous fatty acids in *R. solanacearum* (data not shown), implying that AcpP3 differs from *E. faecalis* AcpB, which does not function in FAS but channels exogenous acyl groups (Zhu et al., 2019). However, like AcpP1, AcpP3 can be acylated by *V. harveyi* AasS, and AcpP3 restored the production of AHL in *P. aeruginosa* mutant strain PA-A1. Therefore, we infer that gene *acpP3* was acquired by horizontal transfer, and AcpP3 cannot interact with FAS proteins of *R. solanacearum* and *E. coli* but of some species such as *P. aeruginosa* and *V. harveyi*. However, the functions of AcpP3 require further investigation.

*Ralstonia solanacearum acpP2* restored the production of xanthomonadins to the *X. campestris xanC* mutant. *acpP2* is located in a large gene cluster in which the genes are highly homologous to those encoded by the *X. campestris pig* gene cluster, although there is no homolog of the *xanB2* gene in *R. solanacearum* (Cao et al., 2018). These data imply that the gene cluster in which *acpP2* is located is involved in the synthesis of a secondary metabolite, similar to the xanthomonadins. *acpP4* also restored the production of xanthomonadins to the *xanC* mutant but is located in another gene cluster, which is similar to the *ape* cluster in *A. baumannii*. In the production of APE, the AcpP4 homolog AbApeE acts as a starter, and the AcpP5 homolog AbApeF forms malonyl-ACP, which provides two carbon units for benzoyl-AbApeE (Grammbitter et al., 2019; Lee et al., 2021). However, in this study, we could not purify the AcpP4 protein or test the functions of AcpP4 and AcpP5 *in vitro*. Therefore, the functions of AcpP4 and AcpP5 require further investigation. Previous studies have shown that five protein residues of *E*. *coli* ACP, Phe28, Phe50, Ile54, Ala59, and Tyr71, affected the interaction of ACP and fatty acyl chain (Roujeinikova et al., 2007). In this study, AcpP2 and AcpP5 could not be acylation *in vitro* (Figure 5C), nor could AcpP2, AcpP4, and AcpP5 involve in FAS *in vivo*. Except because the possible inability of these three ACPs to interact with FAS proteins, it also may be due to these three ACPs cannot covalently attach to the fatty acyl chain, as most of the above five residues of these three ACPs are different from those of *E*. *coli* ACP (Figure 1C).

Single gene *R. solanacearum* mutants lacking *acpP2*, *acpP3*, *acpP4*, or *acpP5* did not affect the pathogenicity of *R. solanacearum*. However, the pathogenicity of the mutant strain mRP2345, which lacked the *acpP2*, *acpP3*, *acpP4*, and *acpP5* genes, was attenuated. This suggests that these *acp* genes encode redundant functions that allow *R. solanacearum* to invade its host or gain a competitive advantage in the soil. However, the functions of these ACPs in *R. solanacearum* require further research.

## Data availability statement

The original contributions presented in this study are included in the article/Supplementary material, further inquiries can be directed to the corresponding authors.

## Author contributions

YY constructed ACP deleted mutants, tested the pathogenicity of mutant strains, and carried out biochemical studies. RL cloned the ACP genes and constructed vectors. W-TL purified ACP proteins and tested the activity of ACP *in vitro*. W-BZ and ZH analyzed fatty acids composition of acpP strains. J-CM participated in the design of the study and helped to draft the manuscript. H-HW conceived of the study, and participated in its design and coordination, and helped to draft the manuscript. All authors read and approved the final manuscript.
