# Supplementary material for: Of its five acyl carrier proteins, only AcpP1 functions in Ralstonia solanacearum fatty acid synthesis
Source: Front Microbiol. 2022 Sep 23;13:1014971. doi: 10.3389/fmicb.2022.1014971 (PMC9542644; doi:10.3389/fmicb.2022.1014971)
Supplement: Supplementary file 1 [file Data_Sheet_1.docx]

Supplementary Material

# Supplementary Tables

## Supplementary Table 1. Bacterial strains and plasmids used in this study

| Strain or plasmid | Relevant characteristic(s) ^a^ | Source or reference |
| --- | --- | --- |
| Strains | | |
| *Escherichia. coli* | | |
| DH5*α* | F^-^ *deoR* *endA1 gyrA96 hsdR17*(r_K_^-^m_K_^+^) *recA1* *relA1* *supE*44 *thi-1*Δ(*lacZYA-argF*)*U*169(φ80*lacZ*ΔM15) | Lab collection |
| BL21(DE3) | F^-^ *dcm ompT* *hsdS* (*r_B_*^-^ *m_B_*^-^) *gal* (λDE3) | Lab collection |
| S17-1 | F^-^ *thi pro* *hsdR* [RP4-2 Tc::Mu Km::Tn7 (Tp Sm)] | Lab collection |
| YY121 | DH5*α* carrying plasmid pJT5, which expressing *E*. *coli* *acpH* gene under *ara*BAD promoter control. | Thomas & Cronan, 2005 |
| CY1877 | *E.coli* MG1655 *acpP*::Cm^r^ carrying pBAD24-*EcacpP* | Lab collection |
| *Ralstonia*. *solanacearum* | | |
| GMI1000 | Wild-type strain; Cm^r^ | ATCC |
| mRP1 | GMI1000 *acpP1*::*EcacpP* | This work |
| mRP1D39V | GMI1000 ∆*acpP1*^D39V^ | This work |
| mRP1I55A | GMI1000 ∆*acpP1*^I55A^ | This work |
| mRP2 | GMI1000 ∆*acpP*2 | This work |
| mRP3 | GMI1000 ∆*acpP3* | This work |
| mRP4 | GMI1000 ∆*acpP4* | This work |
| mRP5 | GMI1000 ∆*acpP5* | This work |
| mRP2345 | GMI1000 ∆*acpP2* ∆*acpP3* ∆*acpP4* ∆*acpP5* | This work |
| *Pseudomonas*. *aeruginosa* | | |
| PAO1 | Wild-type strain; Km^r^ | Lab collection |
| PA-A1 | PAO1 *acpP1*::*EcacpP* | Ma *et al*., 2017 |
| *Xanthomonas*. *campestris* | | |
| XCC8004 | Wild-type strain; Rif^r^ | Lab collection |
| mXPC | Xcc8004 ∆*xanC* | This work |
| Plasmids | | |
| pET-28b(+) | Km^r^; IPTG-inducible expression vector | Novagen |
| pSRK-Km | Km^r^; IPTG-inducible broad-host-range expression vector containing *lac* promoter and *lacI^q^*, *lacZα*^+^ | Khan *et al*., 2008 |
| pSRK-Gm | Gm^r^; IPTG-inducible broad-host-range expression vector containing *lac* promoter and *lacI^q^*, *lacZα*^+^ | Khan *et al*., 2008 |
| pTac85 | Amp^r^; IPTG-inducible expression vector | Marsh, 1986 |
| pK18mobscaB | Km^r^; *sacB*-based gene replacement vector | Schafer *et al*., 1994 |
| pET-EcacpS | *E*. *coli acpS* cloned into the *Nde*I and *Hind*III sites of pET28b(+) | Chen *et al*., 2022 |
| pET-Sfp | *B*. *subtilis sfp* cloned into the *Nde*I and *Hind*III sites of pET28b(+) | Chen *et al*., 2022 |
| pYFJ84 | *V. harveyi aasS* cloned into the *Nde*I and *BamH*I sites of pET16b | Jiang *et al*., 2006 |

^a^ Cm^r^, Km^r^, Rif^r^, Gm^r^, Amp^r^ represent the resistance to chloramphenicol, kanamycin, rifamycin, gentamicin and ampicillin, respectively.

**References**

Chen, Y. C., Hu, Z., Zhang, W. B., Yin, Y., Zhong, C. Y., Mo, W. Y., *et al*. (2022). HetI-like phosphopantetheinyl transferase posttranslationally modifies acyl carrier proteins in *Xanthomonas*. *Mol*. *Plant* *Microbe Interact*. 35(4), 323-335. doi:10.1094/MPMI-10-21-0249-R.

Jiang, Y., Chan, C. H. and Cronan, J. E. (2006). The soluble acyl-acyl carrier protein synthetase of *Vibrio harveyi* B392 is a member of the medium chain acyl-CoA synthetase family. *Biochemistry*. 45(33), 10008-10019. doi: 10.1021/bi060842w

Khan, S. R., Gaines, J., Roop, R. M. II, and Farrand, S. K. (2008). Broad-host-range expression vectors with tightly regulated promoters and their use to examine the influence of TraR and TraM expression on Ti plasmid quorum sensing. *Appl*. *Environ*. *Microbio**l*. 74, 5053-5062. doi: 10.1128/AEM.01098-08

Ma, J. C., Wu, Y. Q., Cao, D., Zhang, W. B. and Wang, H. H. (2017). Only acyl carrier protein 1 (AcpP1) functions in *Pseudomonas aeruginosa* fatty acid synthesis. *Front*. *Microbiol*. 8, 2186. doi: 10.3389/fmicb.2017.02186

Marsh, P. (1986). Ptac-85, an *E*. *coli* vector for expression of non-fusion proteins. *Nucleic Acids Res*. 14, 3603. doi: 10.1093/nar/14.8.3603

Schäfer, A., Tauch, A., Jäger, W., Kalinowski, J., Thierbach, G. and Pühler, A. (1994). Small mobilizable multipurpose cloning vectors derived from the *Escherichia coli* plasmids pK18 and pK19: selection of defined deletions in the chromosome of *Corynebacterium glutamicum*. *Gene*. 145, 69-73. doi: 10.1016/0378-1119(94)90324-7

Thomas, J., and Cronan, J. E. (2005). The enigmatic acyl carrier protein phosphodiesterase of *Escherichia coli*: Genetic and enzymological characterization. *J*. *Biol*. *Chem*. 280, 34675-34683. doi:10.1074/jbc.M505736200

## Supplementary Table 2. Oligonucleotides used in this work

| Name | Sequence (5´ to 3´) |
| --- | --- |
| RT-PCR assay |  |
| A1F | TCGCGGTGCTGGCAAA |
| A1R | GAGGTGAGGAAGTCGTGC |
| A2F | GTCCACCTGCGTCACCAC |
| A2R | CAGTCGATGGCAAGCTTG |
| A3F | CTTTCCACCTCCGCTCC |
| A3R | CGTGACGATCCATTCGTC |
| A4F | GCCACCATGTGCTGCTC |
| A4R | CTGGCCCAGTGCATCGG |
| A5F | GGCGGCATGTACATGGG |
| A5R | GCTCGGCGACGATCTTC |
| A6F | GTGCAACAAGCCATCGA |
| A6R | CGACCGAGTTTCCTACC |
| rsc1053qF | GCCGAAGCTGACATCAAGAAC |
| rsc1053qR | CCATCACCAGCTCAACCGT |
| rsc0434qF | CGGCGAACTGAATCTCGAAG |
| rsc0434qR | GCGCAGTTCGAAGCCGTATT |
| rsp1659qF | CAGCGTCGAGATCGTGATGG |
| rsp1659qR | GCGCGGCTTCGATATAGGT |
| rsp0370qF | CGATCTGGAGGATATGACGCC |
| rsp0370qR | TCGATGGAATCCAGGCCGAT |
| rsp0369qF | AACTCGACCTCGACAGCATC |
| rsp0369qR | GATTGGACCGCGCCGATAA |
| gyrBqF | GACCGAACAGCAGAAACCG |
| gyrBqR | GTCCAGCACCTCGAACACG |
| 16SqF | ACACCGCCCGTCACACCA |
| 16SqR | GTTCCCCTACGGCTACCTT |
| Expression plasmid construction | |
| rsc1053F NdeI | GTCGCATATGGACAACATCGAACAACGC |
| rsc1053F NcoI | gacaCCATGGACAACATCGAACAACGC |
| rsc1053R HindIII | GTACAAGCTTTTACGCCTTGACGTTGGCGC |
| rsc1053R SalI | ATCAGTCGACTTACGCCTTGACGTTGGCGC |
| rsc1053 S37T P2 | ccgtgtcgagcgtgtcagcgccgagatcgtt |
| rsc1053 S37T P3 | cggcgctgacacgctcgacacggttgagctg |
| rsc0434F NdeI | AGGTCATatgaacgcactggaaaaag |
| rsc0434F NcoI | gacaCCATGGGCaacgcactggaaaaag |
| rsc0434R HindIII | GTCAAAGCTTATGCTAGCGCACGCGGT |
| rsc0434R SalI | ATCAGTCGACATGCTAGCGCACGCGGT |
| rsc0434 S41T P2 | ggatatcgatCGTgtcgagcgcgaagcctt |
| rsc0434 S41T P3 | cgcgctcgacACGatcgatatcctggaaatcg |
| rsp1659F NdeI | GCTACATATGAGCAACCCGACCGTCCT |
| rsp1659F NcoI | gacaCCATGGGCAGCAACCCGACCGTC |
| rsp1659R HindIII | ACGCAAGCTTGCAGGCTCATGCGGTCT |
| rsp1659R SalI | ATCAGTCGACGCAGGCTCATGCGGTCT |
| rsp1659 S39T P2 | CGCTGTCGAGCGTATCCGCGCCCAGGTCG |
| rsp1659 S39T P3 | GGGCGCGGATACGCTCGACAGCGTCGAGATC |
| rsp0370F NdeI | GCTACATATGGATTCTTTAAAGCTCG |
| rsp0370F NcoI | gacaCCATGGATTCTTTAAAGCTCG |
| rsp0370R HindIII | ACGCAAGCTTTTATTGGTGGCTCTGGACCA |
| rsc0370R SalI | ATCAGTCGACTTATTGGTGGCTCTGGACCA |
| rsp0370 S42T P2 | ATGAGTCGATCGTATCCAGGCCGATGCCGTT |
| rsp0370 S42T P3 | CGGCCTGGATACGATCGACTCATTGGAAATCGG |
| rsp0369F NdeI | GCTACATATGACCGAAACCGAAATCCT |
| rsp0369F NcoI | gacaCCATGGGCACCGAAACCGAAATCCT |
| rsp0369R HindIII | ACGCAAGCTTTCAGGCTGCGAGCAGCGATT |
| rsc0369R SalI | ATCAGTCGACTCAGGCTGCGAGCAGCGATT |
| rsp0369 S39T P2 | CGGCGTCGATcgtGTCGAGGTCGAGTTCTT |
| rsp0369 S39T P3 | CGACCTCGACacgATCGACGCCGTCGATCT |
| ecAcpP F NdeI | gacaCATATGAGCACTATCGAAGAACG |
| ecAcpP R HindIII | ACTCAAGCTTACGCCTGGTGGCCGTT |
| xc4101F NdeI | gacaCATATGTCCTCGCAAACCGC |
| xc4101R HindIII | ACTCAAGCTTTCAGGTAGTCTTGTTGG |
| Suicide plasmid construction | |
| rsc1053 P1 HindIII | CAGTAAGCTTTCCGATGATGAAGGCGCGT |
| rsc1053 P2 | CGAGCGTAATCGATGGCTTGTTAACCCTCCAGGGAAGT |
| rsc1053 P3 | ACTTCCCTGGAGGGTTAACAAGCCATCGATTACGCTCG |
| rsc1053-ec P2 | CGTTCTTCGATAGTGCTCATTTAACCCTCCAGGGAAGT |
| rsc1053-ec P3 | AACGGCCACCAGGCGTAACAAGCCATCGATTACGCTCG |
| rsc1053 P4 XbaI | GTCATCTAGAATGCATGATGCTGAGGTGCC |
| 1053-ecacpP F | ACTTCCCTGGAGGGTTAAATGAGCACTATCGAAGAACG |
| 1053-ecacpP R | CCAAGAGAAAGACGACGCTTACGCCTGGTGGCCGTT |
| rsc1053 D39V P2 | AtTCgACgGTcaccaagctGTCAGCGCCGAGATCGT |
| rsc1053 D39V P3 | ttggtgACcGTcGAatTaGTGATGGCGTTGGAAGAT |
| rsc1053 I55A P2 | GCcTCcTCaTCgGGtgccTCCATACCGAACTCATCTTC |
| rsc1053 I55A P3 | caCCcGAtGAgGAgGCaGAGAAGATCACCACGGTG |
| rsc0434 P1 HindIII | TAGTAAGCTTATCTGGTAAGCGGCAACGGC |
| rsc0434 P2 | AACCGAGCGTGGCGAAGATCTGGTCCGTCCCGTGCATGT |
| rsc0434 P3 | ACATGCACGGGACGGACCAGATCTTCGCCACGCTCGGTT |
| rsc0434 P4 BamHI | CGTAGGATCCACCGAGGCCATGTTGATGAT |
| rsp1659 P1 HindIII | ACGCAAGCTTCGAAGCCCTGTACCAGCATT |
| rsp1659 P2 | CTGTCCTCATCGAATTCGACGTTGCTCATGGTGAAGGTTC |
| rsp1659 P3 | GAACCTTCACCATGAGCAACGTCGAATTCGATGAGGACAG |
| rsp1659 P4 XbaI | GCTATCTAGATTCGCGCTGGAACGCTTC |
| rsp0370 P1 HindIII | ACGCAAGCTTAAGTGCAGGGTGCATCG |
| rsp0370 P2 | CAGCTGATACTGCTTGCGATCGAGCGTCTCGATCAG |
| rsp0370 P3 | CTGATCGAGACGCTCGATCGCAAGCAGTATCAGCTG |
| rsp0370 P4 XbaI | GCTATCTAGAAGGTCAACAGCCCGGCAA |
| rsp0369 P1 HindIII | ACGCAAGCTTACCGGCTGCATCCGTTCT |
| rsp0369 P2 | AGTTTGATGGCCAGATCGACGTGATGTTCTCCCGTTCCAG |
| rsp0369 P3 | CTGGAACGGGAGAACATCACGTCGATCTGGCCATCAAACT |
| rsp0369 P4 XbaI | GCTATCTAGAATCAGTGCGTTGACGGCA |
| rsp0369-70 P1 HindIII | ACGCAAGCTTAATACGTCAGCAACGCGGAC |
| xcacpC P1 HindIII | ACTCAAGCTTCTGCTCGCCATCGAACG |
| xcacpC P2 | CAGGCCGGTATTGAACAGGCTGAGGGGTGCGGGTAA |
| xcacpC P3 | TTACCCGCACCCCTCAGCCTGTTCAATACCGGCCTG |
| xcacpC P4 BamHI | TGCTGGATCCCGCGTGTAGGTGTATTGC |
| Mutant verification | |
| rsc1053 P5 | GATGGCCGTCATCGAC |
| rsc1053 P6 | GTCGTGCAAGCCGTGA |
| rsc1053 D39V R | tAatTCgACgGTcaccaagct |
| rsc1053 I55A R | tGCcTCcTCaTCgGGtgcc |
| rsc0434 P5 | ATTCGAAATGGCGCGCCA |
| rsc0434 P6 | ATGCCAGGTACGCCACCAT |
| rsp1659 P5 | GCTATCTGCTCGAGCACG |
| rsp1659 P6 | CCTCGGTCGTCAGCAGT |
| rsp0370 P5 | CGTATGCGGGCTGCTGTT |
| rsp0369 P6 | GATGCACAGCGCATAGCG |

Note: underlined letters show restriction sites.

## Supplementary Table 3. Identities between RsACP and other ACP amino acid sequences

| Protein | EcAcpP | XcXanC | AbApeE | AbApeF |
| --- | --- | --- | --- | --- |
| RsAcpP1 | 68.35% | — | — | — |
| RsAcpP2 | — | 48.31% | 33.70% | — |
| RsAcpP3 | 35.63% | — | — | — |
| RsAcpP4 | — | 41.11% | 44.09% | — |
| RsAcpP5 | 29.63% | — | — | 41.46% |

Note: identities ≤ 25% are indicated as “—”.

## Supplementary Table 4. Analysis of fatty acid compositions in R. solanacearum strains^a^

| Fatty acid^b^ | Composition (%) | | |
| --- | --- | --- | --- |
|  | GMI1000 | mRP1 | mRP2345 |
| n-C_14:0_ | 2.84 ± 0.04 | 2.99 ± 0.51 | 2.91 ± 0.33 |
| n-C_14:0_-3-OH | 14.33 ± 1.60 | 14.05 ± 2.05 | 12.08 ± 0.24 |
| n-C_16:1_ | 15.42 ± 0.54 | 16.88 ± 0.91 | 13.01 ± 1.02 |
| n-C_16:0_ | 18.37 ± 1.93 | 23.64 ± 2.90 | 31.67 ± 2.18 |
| n-C_18:1_ | 33.03 ± 2.41 | 29.46 ± 2.44 | 24.90 ± 2.01 |
| n-C_18:0_ | 13.75 ± 1.21 | 12.88 ± 2.07 | 15.43 ± 1.42 |

^a^Cells were grown in BG medium to OD_600_ = 0.7. Total lipids were extracted and transesterified to fatty acid methyl esters, and the products were identified with GC–MS. The values are percentages of the total fatty acids and are the means ± standard deviations of three independent experiments.

^b^n-C_14:0_, tetradecanoic acid; n-C_14:0_-3-OH, 3-hydroxytetradecanoic acid; n-C_16:1_, hexadecenoic acid; n-C_16:0_, hexadecanoic acid; n-C_18:1_, octadecenoic acid; n-C_18:0_, octadecanoic acid.

# Supplementary Figures


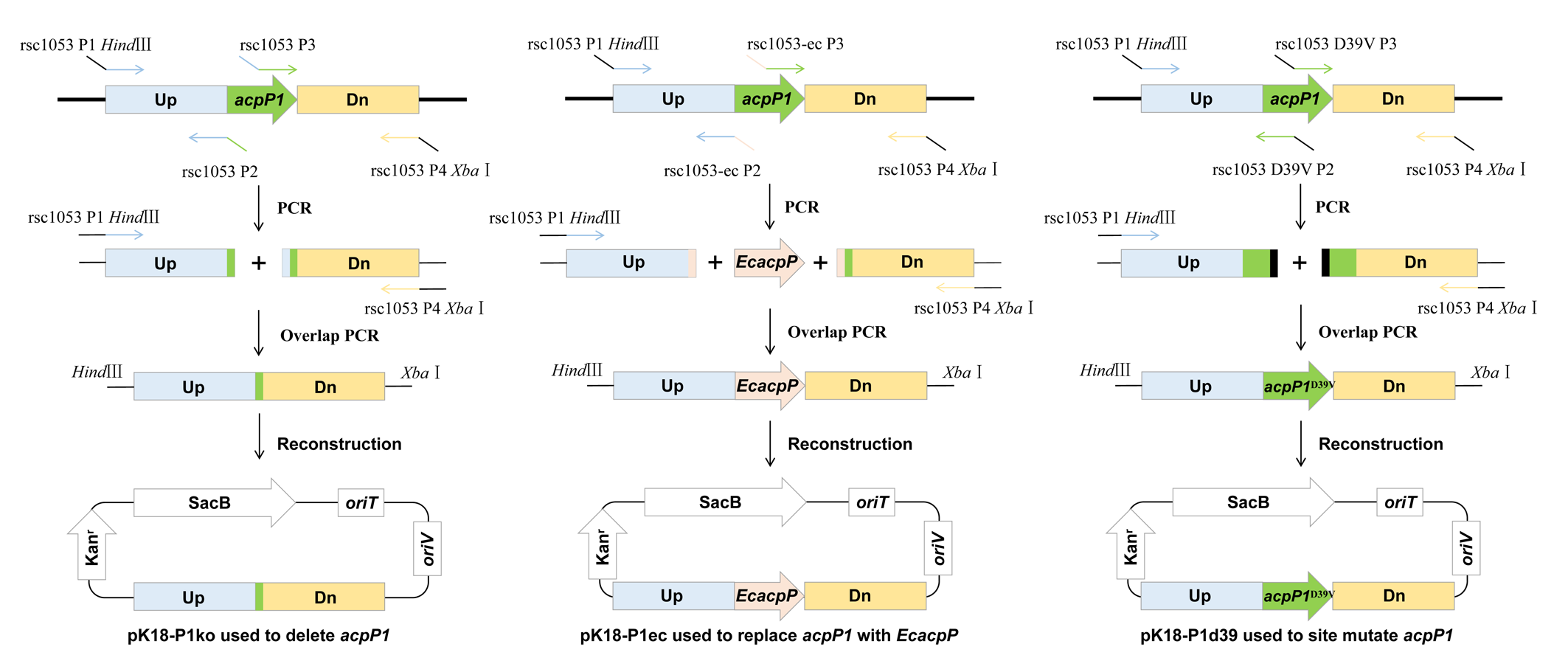


**Supplementary Figure 1.** Three strategies were used to construct pK18mobscaB-derived vectors for *acp* gene mutation. DNA fragments ligated into pK18mobscaB with restriction-enzyme cleavage sites.


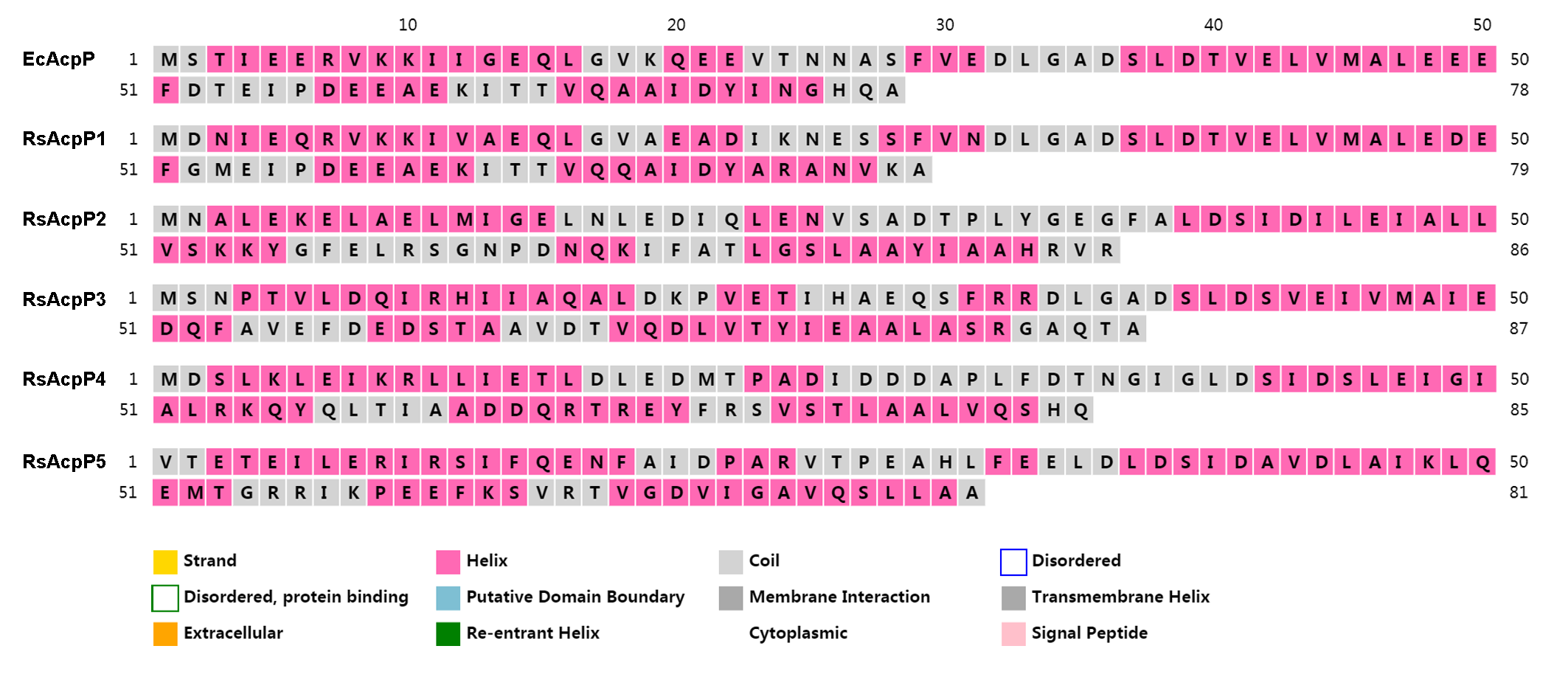


**Supplementary Figure 2.** Secondary-dimensional structures of RsACPs predicted with the PSIPRED server (http://bioinf.cs.ucl.ac.uk/psipred).


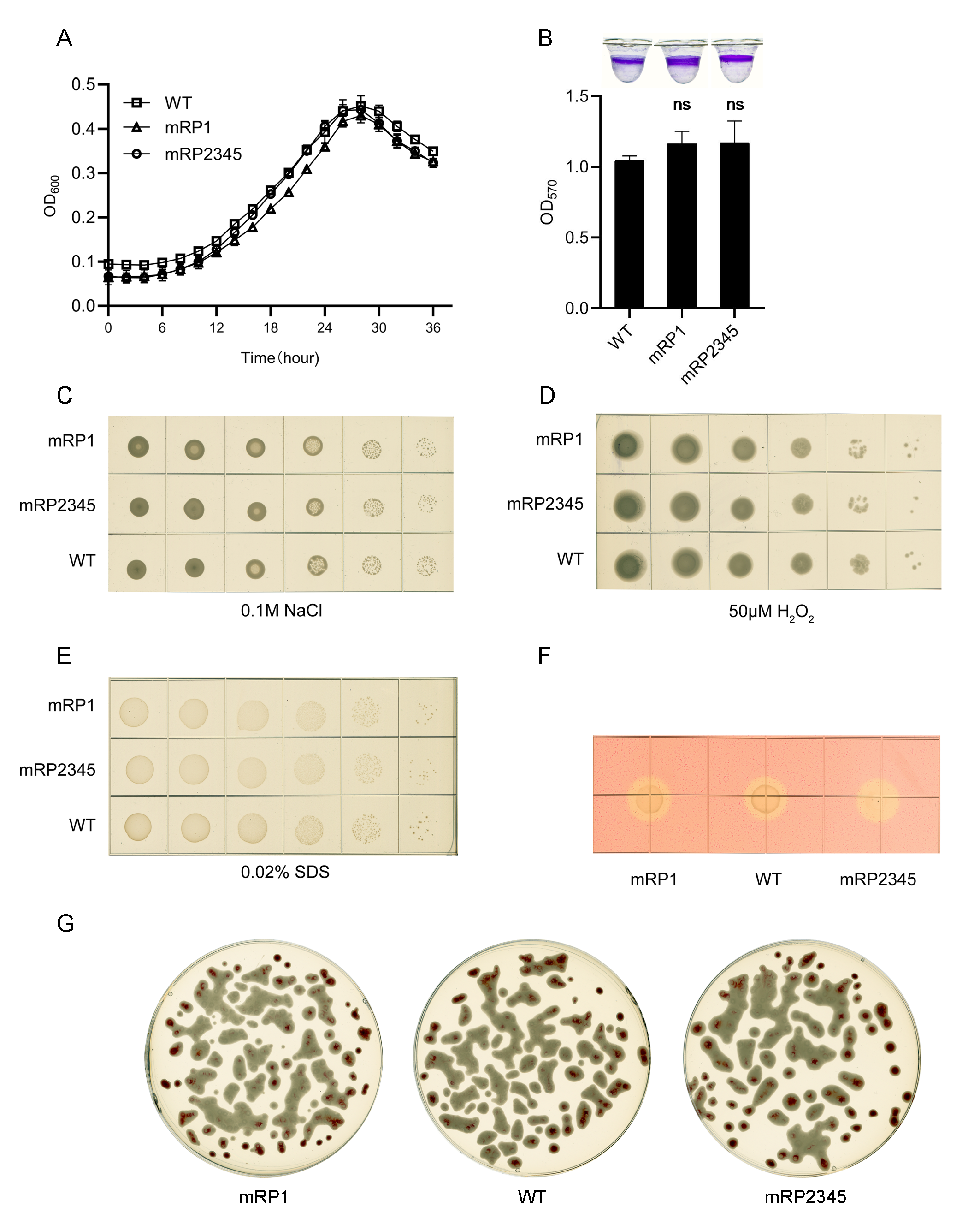


**Supplementary Figure 3.** Properties of *R*. *solanacearum* *acp* mutant strains. (A) Growth of the strains in BG medium. (B) Biofilm formation of strains cultured in BG medium. (C) Strains cultured in 1/10 BG medium containing (a) 0.1 M NaCl, (b) 50 μM H_2_O_2_, or (c) 0.02% SDS. (D) Detection of extracellular cellulase production by *R*. *solanacearum* strains on 1/10 BG medium plus 0.2% carboxymethyl cellulose. (E) Production of extracellular polysaccharides by *R. solanacearum* strains on BG medium plus 0.05‰ triphenyltetrazolium chloride.
